# Just-in-Time, but Still Planned: Lessons Learned From Speeding up the Development and Implementation of an Intervention to Promote COVID-19 Vaccination in University Students

**DOI:** 10.1177/15248399221095077

**Published:** 2022-05-22

**Authors:** Gill A. ten Hoor, Tugce Varol, Ilse Mesters, Francine Schneider, Gerjo Kok, Robert A. C. Ruiter

**Affiliations:** 1 Maastricht University, Maastricht, The Netherlands

**Keywords:** COVID-19 vaccination, Intervention Mapping, time lags, intervention development, health promotion

## Abstract

The process of developing a behavior change intervention can cover a long time period. However, in times of need, this development process has to be more efficient and without losing the scientific rigor. In this article, we describe the just-in-time, planned development of an online intervention in the field of higher education, promoting COVID-19 vaccination among university students, just before they were eligible for being vaccinated. We demonstrate how intervention development can happen fast but with sufficient empirical and theoretical support. In the developmental process, Intervention Mapping (IM) helped with decision-making in every step. We learned that the whole process is primarily depending on the trust of those in charge in the quality of the program developers. Moreover, it is about applying theory, not about theory-testing. As there was no COVID-19-related evidence available, evidence from related fields helped as did theoretical knowledge about change processes, next to having easy access to the target population and important stakeholders for informed qualitative and quantitative research. This project was executed under unavoidable time pressure. IM helped us with systematically developing an intervention, just-in-time to positively affect vaccine acceptance among university students.

COVID-19 caused many problems and forced health promoters to develop interventions under unavoidable time pressure. This haste is challenging as on average it takes 17 years “to move medical research from bench to bedside” (Morris et al., 2011, p. 510). However, the scientific process can become more efficient in times of need, and without losing credibility (Hanney et al., 2015). Especially, the COVID-19 pandemic taught us that there are ways to speed up intervention development and implementation, without losing scientific rigor (Hanney et al., 2020).

In this article, we describe the planned development of an online intervention to promote COVID-19 vaccination among students at Maastricht University (The Netherlands) within a time frame that was necessarily much shorter than usual (see Figure 1) because the age group of the students was eligible for vaccination within 6 months. Hanney et al. (2020) formulated four overlapping strategies to shorten the time lags from problem identification to intervention (or program) implementation in practice: (1) increasing resources (e.g., funding), (2) working in parallel (e.g., starting a next step if there is enough information), (3) starting or working at risk (e.g., expert consensus instead of new research), and (4) improving processes (e.g., accelerating procedures).

**Figure 1:**
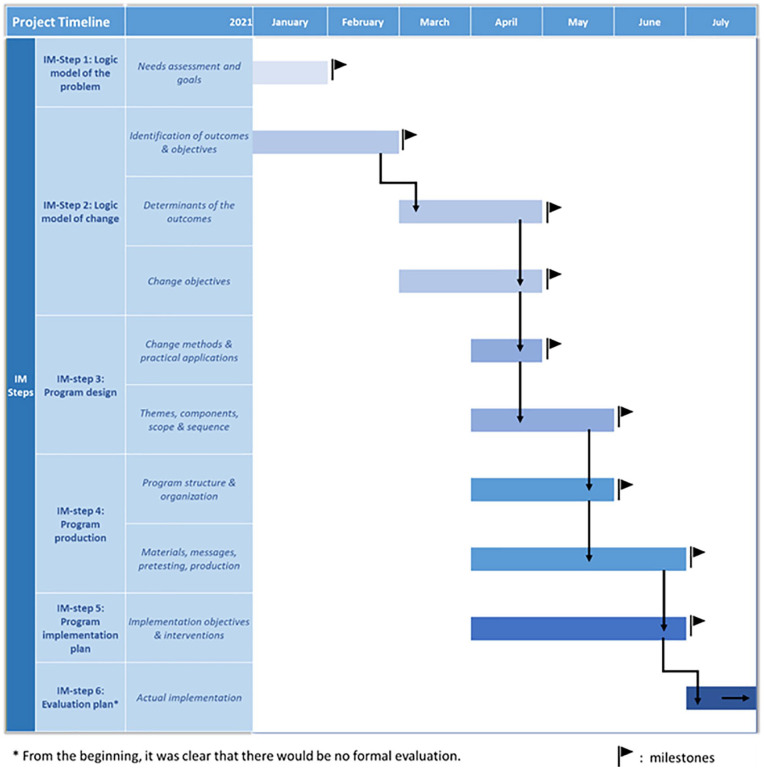
Time Frame *Note.* IM = Intervention Mapping.

In the current project (and in line with Hanney’s suggestions), the importance of a high vaccination coverage was recognized by the University’s leadership as a condition for a safe reopening of the facilities, and for on-site teaching. Therefore—reducing further delays in the intervention development—resources were made available to facilitate our iterative intervention development (in line with the suggestions of Kwasnicka et al. (2021)). To further optimize efficiency and reducing time lags, several decisions were either based on psychological theories (e.g., reasoned action approach, when empirical evidence was not available) or taken in parallel/simultaneously by different stakeholders (e.g., research team, video/website developers, university board). With that, automatically more risks were taken in terms of (mis)communication, (faulty) decisions during the process, subsequent (in)effectiveness of the intervention, and with that (lowered) cost-effectiveness. To improve the intervention development process, and to limit the financial and safety risks, we applied the six steps of the Intervention Mapping (IM) protocol (Bartholomew Eldredge et al., 2016; Fernandez, Ruiter, et al., 2019; Kok et al., 2016). IM is a protocol that guides the design of multilevel health promotion interventions and implementation strategies (Bartholomew Eldredge et al., 2016). IM consists of six steps: (1) conduct a needs assessment or problem analysis by identifying what, if anything, needs to be changed and for whom; (2) create matrices of change objectives by crossing performance objectives (sub-behaviors) with determinants; (3) select theory-based intervention methods that match the determinants, and translate these into strategies, or applications, that satisfy the parameters for effectiveness of the selected methods; (4) integrate the strategies into an organized program; (5) plan for adoption, implementation, and sustainability of the program in real-life contexts by identifying program users and supporters and determining what their needs are and how these should be fulfilled; (6) generate an evaluation plan to conduct effect and process evaluations to measure program effectiveness. Essentially, Steps 1 to 4 focus on the development of multilevel interventions to improve health behaviors and environmental conditions, Step 5 focuses on the development of implementation strategies to enhance program use, and Step 6 is used to plan the evaluation of both the program itself and its implementation. Within each step of IM, the so-called “Core Processes” (Ruiter & Crutzen, 2020) were used to identify the important literature, apply the appropriate theories, and collect essential additional research data. In the following section, we will describe the IM steps that we took in more detail. In the Discussion section, we will reflect on the process in more detail in relation to the four strategies of Hanney et al. (2020).

## IM-STEP 1: Logic model of the problem

COVID-19 is a new infectious disease (Ciotti et al., 2020). Its severity is highly variable, ranging from unnoticeable to life-threatening. Severe illness is more likely in elderly COVID-19 patients, as well as those who have underlying medical conditions. COVID-19 may transmit when people breathe in air contaminated by droplets and small airborne particles. People may spread the virus even if they do not develop any symptoms. Preventive measures reducing the chances of infection include, also for students: getting vaccinated, staying at home, wearing a mask in public, avoiding crowded places, keeping distance from others, ventilating indoor spaces, managing potential exposure durations, washing hands with soap and water often and for at least 20 seconds. Moreover, COVID-19 vaccines have demonstrated efficacy as high as 95% in preventing COVID-19 infections. At that time, in the Netherlands, those not vaccinated made up the large majority of COVID-19 patients (80%–90%), and vaccination coverage was around 85% in the adult population (Rijksinstituut voor Volksgezondheid en Milieu, 2021). Several vaccines have been developed and widely distributed since December 2020 (World Health Organization, 2020). Therefore, the *goal* of our program was to promote vaccination acceptance among university students, within a setting of informed decision-making which characterizes the approach of the Dutch government in motivating people to participate in national vaccination programs: “Given the availability of confusing and conflicting vaccine narratives, it is crucial that authoritative communication materials aim to build trust and support informed choices about vaccination” (Vivion et al., 2020, p. 112).

**Table:** 

***Just-in-time*:** This step could be taken quite fast, as almost all information was already easily available.

## IM-STEP 2: The logic model of change

### Identification of Behavioral and Environmental Outcomes and Performance Objectives

In the first half of 2021, everyone aged 18 years and over in the Netherlands was, or would be, invited to be vaccinated against COVID-19, which is considered a voluntary decision (Government of the Netherlands, 2022). Visiting international students could be vaccinated as well, and the University has an agreement with the Local Public Health Office to provide those vaccinations. The *behavioral outcome* for all students in this case is responding positively to the invitation for the vaccination or, when a visiting international student, following up on the offer to contact the Local Public Health Office. For the University, the *environmental outcome* is limited to informing incoming international students among the whole student population about the existing facilities for vaccination. The behavioral outcome is relatively easy achievable as long as people have a positive intention, as there are few barriers (daCosta DiBonaventura & Chapman, 2005; Fall et al., 2018). For students, the *performance objectives*—what do the participants in the program need to do to perform the behavioral outcome?—include: scheduling the vaccination appointment, remembering to go, preparing all necessary paperwork, and following instructions on time, place, and optimal preparation (e.g., clothing, forms, and identification). The environmental outcomes and performance objectives for the Local Public Health Service are already in place.

### Determinants of the Behavioral Outcomes

At that time, there were no systematic reviews of determinants for COVID-19 vaccination in university students. Our earlier articles in the same setting described the qualitative and quantitative studies among students about (social) preventive behaviors (e.g., distancing, testing), including a short intervention to promote preventive behaviors when students go home for the Christmas/New Year holiday (Varol et al., 2021a, 2021b). A third study, a cross-sectional online survey with the University students’ panel (*N* = 434) on vaccination behavior, was conducted in March 2021 (Varol et al., 2022). Given the need for fast development, we formulated our questions based on existing validated theoretical constructs (e.g., Fishbein & Ajzen, 2010). Also, the existence of an ongoing student panel was a great advantage. We explored university students’ intentions to be vaccinated and selected the most relevant *determinants* and their underlying beliefs to facilitate informed decision-making around COVID-19 vaccine uptake. We found that students’ intention to be vaccinated is high (80% positive). Concerns about safety and side effects of the vaccine and trust in government, quality control, and the pharmaceutical industry were identified as the most relevant determinants of vaccine intention (e.g., “I trust the quality control around the COVID-19 vaccine” or “I am worried about the safety of the COVID-19 vaccine”). Other predictors are risk perception (e.g., “I think that without vaccination, I might be at risk of contracting COVID-19”), attitude (e.g., “I think that getting the COVID-19 vaccine is a way out of this pandemic”), perceived norm (e.g., “I think that most people who are important to me want me to get the COVID-19 vaccination”), and self-efficacy beliefs (e.g., “I am confident that before I decide to get the COVID-19 vaccine, I will have sufficient information about the COVID-19 vaccine”).

### Change Objectives

Change objectives are constructed by combining performance objectives with determinants; they form the most proximal intervention targets. Examples of change objectives are in this case: “Students state that they are not worried about the safety of the COVID-19 vaccine,” “Students recognize that their doctor/health care provider wants them to get the COVID-19 vaccination,” or “Students indicate that it is easy for them to get the COVID-19 vaccine when it is their turn.” In Table 1 (Varol et al., 2022), the selected change objectives are listed in the first column. Except for two change objectives about “concerns” (that are negative and supposed to decrease), all these objectives are positively formulated and are targeted for improvement (second column) as they were all correlated with the vaccination intention, and there was still room for improvement in those beliefs.

**Table:** 

***Just-in-time*:** Step 2 needed empirical studies into the determinants of students’ vaccination intentions. The protocol for those kinds of study is clearly explained in the IM process. As the University already had a student panel, the study could be executed quite fast, helped by efficient decision-making at the level of the University management.

## IM-STEP 3: program design

### Theory- and Evidence-Based Change Methods and Practical Applications

In Table 1, the change objectives are linked to theory- and evidence-based change methods (third column). A *change method* is a defined process by which theories postulate, and empirical research provides evidence for, how change may occur: “a general technique for influencing the determinants of behaviors and environmental conditions.” In our case, we selected the change methods based on those as formulated by Bartholomew Eldredge et al. (2016, p. 347). An *application* is a way of organizing, operationalizing, and delivering the intervention methods: “delivery of the methods in ways that fit the intervention population and the context in which the intervention will be conducted” (p. 347). Translating methods into applications demands a sufficient understanding of the theory behind the method, that is the theoretical *parameters* that are necessary for the effectiveness of the theoretical process of change (fourth column in Table 1). For example, consciousness-raising may increase risk perception, but only when people have the skills and self-efficacy to counter the risk. Also, information about others’ approval may be highly influential, but only when those others indeed approve of the COVID-19 vaccination. All theoretical methods have these parameters and those need to be taken into account when the method is applied in real life.

**Table 1.** Examples of Pairing the Relevant Determinant With Behavior Change Methods to Target In Intervention (Varol et al., 2022)

Determinant/item (scale: 1 – 7)	Change direction	Method	Parameters
Risk perception			
Without vaccination, I might be at risk of contracting COVID-19	5.7 ↑	[Belief selection: done] Consciousness raising Framing Self-affirmation	Self-efficacy improvement Gain frames Tailored to the individual
If I contract COVID-19, the physical consequences for me would be severe	3.3 ↑		
If I contract COVID-19, the social consequences for me would be severe	4.4 ↑		
Concerns and Trust			
Concerns about the safety of the COVID-19 vaccine	3.3 ↓	Scenario-based risk info Persuasive communication Tailoring	Plausible cause-effect Relevant, not-discrepant, arguments Interactive (if possible?)
Concerns about possible long-term negative side effects of the COVID-19 vaccine	3.9 ↓		
The COVID-19 vaccine will be effective against new mutations of the virus	3.9 ↑		
I trust the government about ensuring the safety of the COVID-19 vaccine	5.0 ↑		
I trust the quality control around the COVID-19 vaccine	5.5 ↑		
I trust the pharmaceutical industry about the safety of the COVID-19 vaccine	4.7 ↑		
[Compared to current vaccines in the National Immunization Program:]			
I consider the COVID-19 vaccine equally safe	5.0 ↑		
I consider the COVID-19 vaccine equally effective	4.9 ↑		
I consider the COVID-19 vaccine equally trusted	4.5 ↑		
Attitude/Outcome Expectations			
By getting the COVID-19 vaccine, I can safely have more social contacts	5.3 ↑	Shifting focus Self-reevaluation Anticipated regret	New reason (postponed reward) Self-image/high self-efficacy Imagery/positive about avoiding negative consequences
I think that getting the COVID-19 vaccine is my moral duty	5.4 ↑		
I would feel guilty if I transmitted the virus if I decided not to get the vaccine	5.9 ↑		
Perceived Norm/Social Influence			
People like me will get the COVID-19 vaccination	5.5 ↑	Info about others’ approval Resistance to social pressure Mobilizing social support Modeling	Are present Commitment/values Available; trust Reinforcement, identification, self-efficacy, coping
My doctor/health care provider wants me to get the COVID-19 vaccination	5.4 ↑		
People who are important to me want me to get the COVID-19 vaccination	5.6 ↑		
Self-Efficacy/Perceived Control			
I am confident that I could get it when it is my turn	5.7 ↑	Modeling Guided practice Planning coping responses Goal setting	Reinforcement, identification, self-efficacy, coping Demonstration, instruction, enactment Identification and practice Commitment to the goal
It is easy for me to get the COVID-19 vaccine when it is my turn	5.5 ↑		
I will have sufficient information about the COVID-19 vaccine	5.4 ↑		
I can always ask for more information from my general practitioner/PHS	5.6 ↑		
I am not concerned about possible local pain that could occur	5.4 ↑		
From the University			
	Advocacy/active support Technical assistance Mass-media role modeling		Matching style, consciousness raising/persuasion /approval Fit culture and resources Appropriate models being reinforced

### Program Themes, Components, Scope, and Sequence

Earlier (Varol et al., 2022), students indicated that they preferred science-based information from content experts, supported by high-level scientific publications, and not influenced by the pharmaceutical industry. Considering the important change objectives, the selected behavior change methods, and the parameters for effectiveness, the actual intervention existed of a series of videos on a special webpage of the University on COVID-19 directed at students. The final intervention included a series of four interviews, each with a student asking questions to an expert. The first two interviews were about risk perception and worries and trust, with two experts in clinical microbiology, and the second two were on attitudes and perceived norms with two experts in health promotion/health psychology. The third part about perceived control was covered with clear online instructions on how, where, and when to get the COVID-19 vaccine, especially targeting international students. Students also indicated that they wanted information about COVID-19 via emails pointing out information on the University’s website (Varol et al., 2022). At all times, we made sure that the content of the videos (Table 2) covered all identified determinants (Table 1).

**Table:** 

***Just-in-time*:** In Step 2, the information became available on the determinants of vaccination intentions, as well as the ways students preferred to be informed. For Step 3, the whole process of analyzing determinants, choosing methods, applying parameters, and producing applications was made easier by following the IM tasks specified for Step 3.

**Table 2:** Content of the Questions That Were Asked of the Four Experts on Video

*These topics are covered in the interviews with the Maastricht University Medical Center experts*:
1. Risk for self and others—consequences for self and others Most young people do not experience severe consequences from COVID-19, why should I bother? If I have already had COVID-19, do I still need to get vaccinated against COVID-19? How long will the COVID-19 vaccines provide protection? How well do vaccines prevent people from spreading the virus to others even if you do not have symptoms? How effective are the current vaccines against new variants/mutations? 2. Safety and trust—long term and side effects, trust, mutations, quality control How do we know that the vaccines are safe? How good is the quality control? What about side effects and what about long-term side effects? Can we trust the pharmaceutical industry? 3. Easy vs practical difficulties How easy is it to be vaccinated? → Refer to the local Public Health Service
*The next topics are covered in the interviews with the health promotion experts*:
4. Reasons Could you tell us about the main advantage of vaccination? Why would I take the vaccination? At this point, we see that more and more people have been vaccinated—also older people and people from at risk groups. Is it then for students still needed to be vaccinated? Why? If I take the vaccination, can I safely get back to normal have more social contacts? (in the long turn) 5. Perceived norm I have friends who do not want to take the vaccination Are Maastricht University students willing to be vaccinated?—These are of course promising numbers. However, they are numbers. Could you also share some personal stories with us—for instance, of colleagues or students that were vaccinated? Did you get vaccinated yourself and why? And what would be your advice for students?

## IM-STEP 4: Program production

In IM-STEP 4, the program structure and organization, materials, messages, pretesting and production are discussed. The interviews with experts from the University in the areas of vaccination and health promotion are the central element in the program. The video part of the program production was executed by the University’s Video team, a semi-professional group of students that produce video components for the University’s communication department; the input of these students also served as a simplified pretest of the program. The content of the questions asked by a student to the experts in the interviews was derived from the results of the earlier study on determinants (Table 1) and the intervention was in line with the results of the qualitative part of the determinants’ study: all interviewees were introduced as experts in their scientific field (see Table 2).

**Figure 2:**
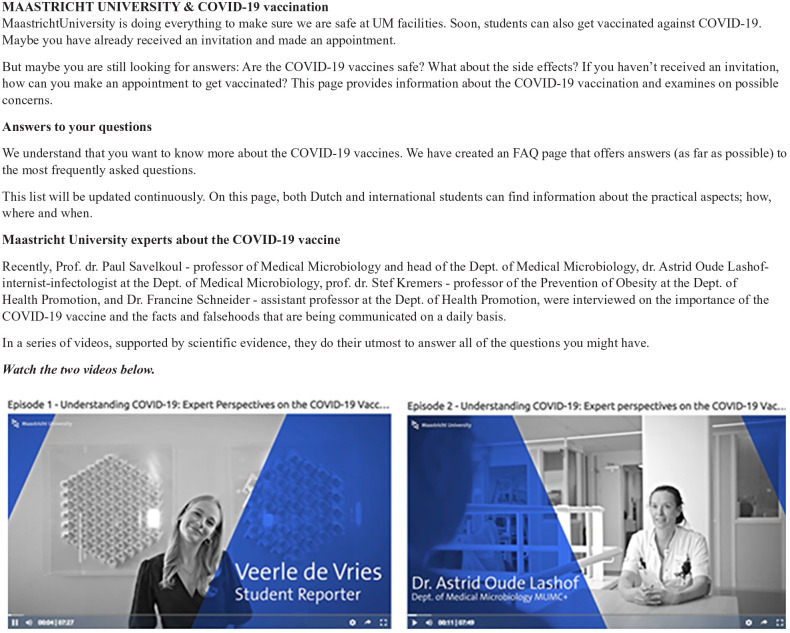
The Maastricht University (UM) and COVID-19 Vaccination Webpage

The final program was a special COVID-19 webpage on the University’s website: https://www.maastrichtuniversity.nl/um-covid-19. Students proceeded to: https://www.maastrichtuniversity.nl/study-safely-during-corona-crisis-1. There they could watch the developed vides: https://maastrichtuniversity.bbvms.com/p/um/c/4327983.html?inheritDimensions=true and https://maastrichtuniversity.bbvms.com/p/um/c/4336725.html?inheritDimensions=true (see Figure 2).

Next to the newly developed videos, there were a series of videos from the “University of the Netherlands” on COVID-19. As those videos were in Dutch, they had been subtitled in English. These videos contained the same information by an expert but are also illustrated by clear animations.

**Table:** 

***Just-in-time*:** The actual intervention could immediately be developed without any time lag, as the communication channels, experts from the Hospital and the University, and video producers were already available.

## IM-STEP 5: PROGRAM IMPLEMENTATION PLAN

In IM-STEP 5, adopters, implementers and maintainers are identified, implementation objectives are stated, and implementation interventions are designed. Implementation is essential for reaching the objectives of an intervention (Fernandez, ten Hoor, et al., 2019). Nevertheless, implementation is often an undervalued aspect of intervention planning as projects have a high chance to run into problems of nonimplementation or under-implementation. However, in this case, from the start, the intervention plan was approved and adopted by the leadership of the university. In collaboration with the University’s Marketing and Communication Department, all services were provided to optimize timely implementation at the start of the summer holidays, just before that age group was eligible for being vaccinated.

**Table:** 

***Just-in-time*:** All facilities for implementation were present and the necessary decision-making processes were followed without any time lag.

## IM-STEP 6: EVALUATION PLAN

Ideally, first-time interventions are systematically developed and evaluated before they go out in the world. However, in times of COVID-19 where further delays were not desired, the systematic evaluation was deliberately skipped. This intervention was based on theory, on the expertise of the authors and communication professionals involved, and was the result of a fast, and just-in-time but still planned process of multidisciplinary inputs with strict timelines. The intervention was made public from the start. The number of views is registered and, knowing that this intervention has an expiration date and that the situation will change, new interventions may be needed.

## Discussion

Evidence-based health promotion interventions are usually developed by applying a systematic process of setting goals and objectives, using research, applying theoretical insights, and collecting data to confirm assumptions. However, in times of need, that process takes too long. Following the suggestions by Hanney et al. (2020), increasing resources, working in parallel, starting or working at risk, and improving processes, the scientific process became shorter. By using IM as a protocol, we made sure that the essential decisions were made in the right order while still using theory and research as optimal as possible. In the following section, we will discuss our lessons learned from implementing the IM protocol.

### Lesson 1: Build a Mutual Trust Relationship Between Relevant Stakeholders and Implementers

The whole process is depending on the trust of those in charge (in this case the leadership of the University) in the competency of the developers. For decision-makers: make sure to include people whose track record you know and who you trust. For implementers: make sure that the people in charge know your expertise in theory- and evidence-based intervention development and implementation.

#### Lesson 2: Make Use of Theory and Core Processes

Theory-testing is not part of this process; this is all about applying theory in a problem-driven context. Especially when time is limited, and therefore research is not always possible, applying theories is the best alternative. One way to systematically apply theories is described in the so-called Core Processes (Ruiter & Crutzen, 2020): (1) pose questions, (2) brainstorm answers, (3) review research, (4) find theoretical support, (5) find empirical support, and (6) complete the list of answers. In Step 4, the planners search for theories, first to understand and then to solve the problem. Core Processes provide a protocol for finding the empirical support and theoretical support that help to quickly formulate appropriate answers to planning questions.

#### Lesson 3: Apply IM

IM helps with detailed note-taking of the decision-making process in intervention development and design, for example what is the risky and what is the safe behavior, what environmental conditions contribute to the problem, who are responsible, what are the determinants of behavior, how can we change those determinants in the desired direction by an intervention, how can we implement the change program, and how can we measure the final outcomes?

#### Lesson 4: Make Use of Evidence From Related Fields

If there is a lack of evidence around the problem, it can be helpful to rely on evidence from related or comparable fields. For example, in Step 3 of the IM process described earlier, the review of empirical findings from published research was limited to articles on other comparable infectious diseases and vaccination programs, such as with influenza or measles, as relevant articles on COVID-19 were not yet available. As a result, the careful application of relevant theories, in a setting of group discussions with experts, formed the basis for “theory- and evidence-based” program development.

#### Lesson 5: Make Use of Evidence From the Past and the Present

Several theory-informed methods (and their parameters for effective application) are identified in the past (Bartholomew Eldredge et al., 2016) that could form the basis of interventions. Here, the identified outcomes, performance objectives, determinants, and change objectives for COVID-19 vaccination acceptance were based on theory and a present survey among the students. This survey provided adequate information about concerns and trust, risk perception, attitudinal beliefs, perceived norms, and self-efficacy beliefs to select the relevant change objectives for the intervention (Table 1). Subsequently, these were linked to the intervention application(s), derived from the earlier identified theory-based methods (Table 1). Given the setting, the target population, and the needs, in IM-Step 3 (program design) an online intervention was chosen as the most efficient way to reach the students.

#### Lesson 6: Identify and Involve All Relevant Stakeholders

It is helpful to identify and involve all stakeholders related to the problem (in this case university students) and solution (experts)—throughout the entire process of intervention development. The focus of the intervention was on science-based information which the students had indicated as the most trustworthy and informative. Therefore, in IM-Step 4, the program design, the major element consisted of four interviews, each with an expert from our own university or academic hospital, discussing the medical aspects: risk for self and others, safety and trust, such as mutations and side effects, and the societal aspects: reasons for taking the vaccination and the interaction with the social environment. We deliberately had a “student asking questions of the experts,” as a voice of all other students. Next to that, the website provides general information about COVID-19 and information about the arrangements at the University for studying in times of COVID-19.

#### Lesson 7: Implementation Can Be More Urgent Than Evaluation or Effect Measures

Often, when there is no time for a randomized controlled study to test the intervention, implementation takes precedence. In this case, the implementation plan was relatively easy, as the University was very helpful and provided all necessary support. The intervention was implemented as soon as it was finished, to promote that students would respond positively to the vaccination invitations that were sent out at that moment in time. IM-Step 6, the evaluation plan, was not executed as the focus was on the moment, and even 1 year later the situation could have changed to a future where everything could be different (e.g., new variants of the virus) and new interventions would be needed.

## Conclusion

The COVID-19 crisis teaches us that there are ways to speed up intervention development and implementation, without losing scientific rigor. The current project was executed under unavoidable time pressure. Nevertheless, IM provided a structure and a process that helped us develop an intervention that hopefully will positively affect students’ vaccination behavior in times of need. We also applied Hanney et al.’s (2020) suggestion about the four ways to speed up the development and implementation of an intervention. For our intervention, *increasing resources* involved (1) concrete support from the University and the National Institute for Public Health, (2) funding of the survey among students, and (3) fast and full implementation of the intervention. *Working in parallel* involved: overlap of the IM-steps as indicated in Figure 1. *Working at risk* involved (1) using evidence from related fields, (2) applying theories to new processes, (3) deciding by expert consensus, and (4) implementing an intervention without evidence for effectiveness. *Improving processes* involved (1) accelerating procedures, (2) using an existing panel of students, (3) collaborating intensively with the department of Marketing and Communication, and (4) following the IM protocol as efficiently as possible. IM was a helpful guide to ensure scientific rigor and quality, while shortening the time between research and application, creating a just-in-time but still planned theory- and evidence-based intervention.
